# Congenital spinal dysraphism with infected sacrococcygeal sinus tract: need for improved awareness amongst clinicians

**Published:** 2020

**Authors:** Shameem AHMED, Deep DUTTA, Siba Prosad PAUL

**Affiliations:** 1 Senior Consultant Neurosurgeon, Department of Neurosurgery, Apollo Hospitals, (Unit: International Hospital), Guwahati, India.; 2 Consultant Neurosurgeon,Department of Neurosurgery, Apollo Hospitals, (Unit: International Hospital), Guwahati, India.; 3 Consultant Paediatrician, Department of Paediatrics, Torbay Hospital, Torquay, UK.

**Keywords:** Spinal dysraphism, Recurrent infections, Sinus tract, Magnetic resonance imaging, Spina bifida occultaIntroduction

## Abstract

Spinal dysraphism (SD) includes a group of developmental anomalies resulting from failure of fusion of parts along dorsal aspect of midline structures lying along spinal axis from skin to vertebrae and spinal cord. There are two types of SD, open and closed. Close SD, also known as spina bifida occulta, can present with diagnostic challenges in resource limited settings where awareness regarding the condition and specialist radiological investigations, including Magnetic Resonance Imaging (MRI), may not be easily available. Undiagnosed cases can potentially lead to long term morbidities. We report the case of a 13-year old boy with closed SD presenting with recurrent infections of the sacrococcygeal sinus tract which were treated with oral antibiotics for what was considered to be localized infection. Following neurosurgical assessment and spinal MRI a diagnosis of SD was made. He underwent surgical excision of the sinus tract and closure of the defect with good outcome. The case emphasizes the need for awareness regarding SD in children who have sinus tracts in the intergluteal fold with symptoms of recurrent discharge and infection.

Spinal dysraphism (SD) occurs when there are developmental abnormalities affecting the dorsal median region in embryo, variably involving ectoderm, mesoderm and endoderm, and includes all forms of spina bifida.^1^ There are two types of SD: spina bifida occulta, also known as closed SD, and open SD also called spina bifida cystica/aperta.^1^ Magnetic resonance imaging (MRI) is currently the investigation of choice for diagnosing spinal dysraphism and for planning surgical management for SD and associated syndromes including Arnold-Chiari malformation or caudal regression syndrome.^2^ This case study describes a 13 years old boy with spina bifida occult SD (OSD) presenting with recurrent infection of sacrococcygeal sinus tract.

## Case Report

**Figure 1:**
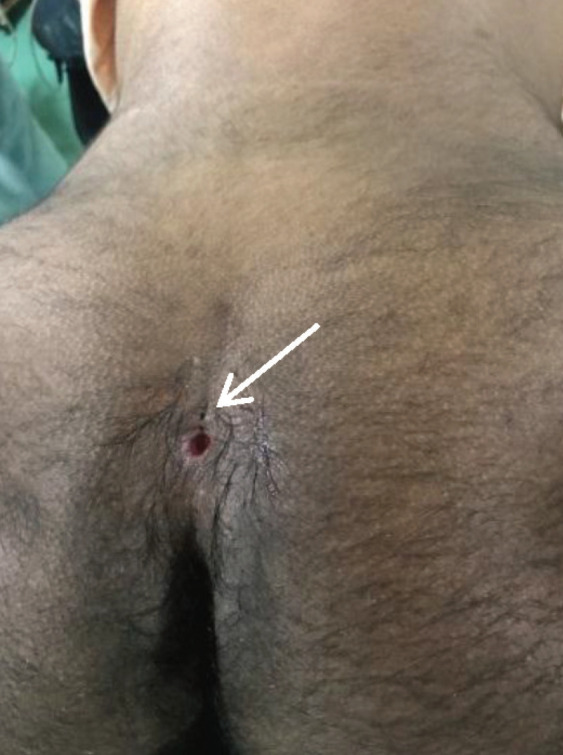
Photograph of the lesion at presentation

A 13-year-old boy presented to the neurosurgical unit in a specialist hospital in north-eastern India with history of a discharging sinus in the sacrococcygeal region (Figure 1). His parents reported that from early infancy the boy had occasional discharge from the area which intermittently became red and painful. These episodes were treated with oral antibiotics by a local physician. He was reported to be doing well both at school and in sports. Parents felt it was necessary to seek specialist advice as his condition was not improving and was affecting his social life and emotional wellbeing. 

Assessment in the neurosurgical clinic revealed normal growth and development; neurological and systemic examination were also normal except for the lesion shown in Figure 1. MRI scan of the lumbosacral spine showed a T2 and Stir hyperintense midline sinus tract, with length of approximately 57 mm and maximum diameter of approximately 8 mm, arising from the lower sacral region and extending at approximately 8 mm from the skin with no identifiable external opening; there was no other abnormality. A diagnosis of SD with sinus tract was made and the family was counselled for surgery; it was explained that recurrent infective episodes were likely to be due to faecal soiling or blockage of the sinus tract by discharge.

**Figure 2 and 3:**
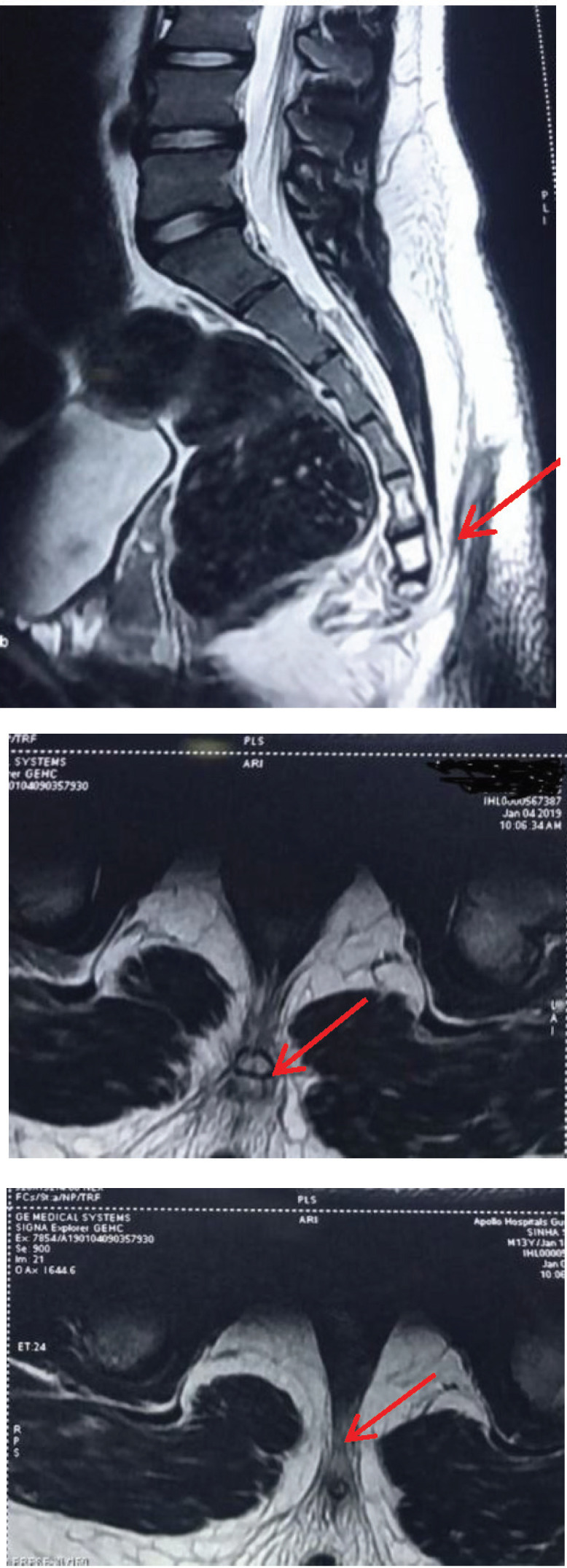
MRI images showing SD

**Figure 4 and 5:**
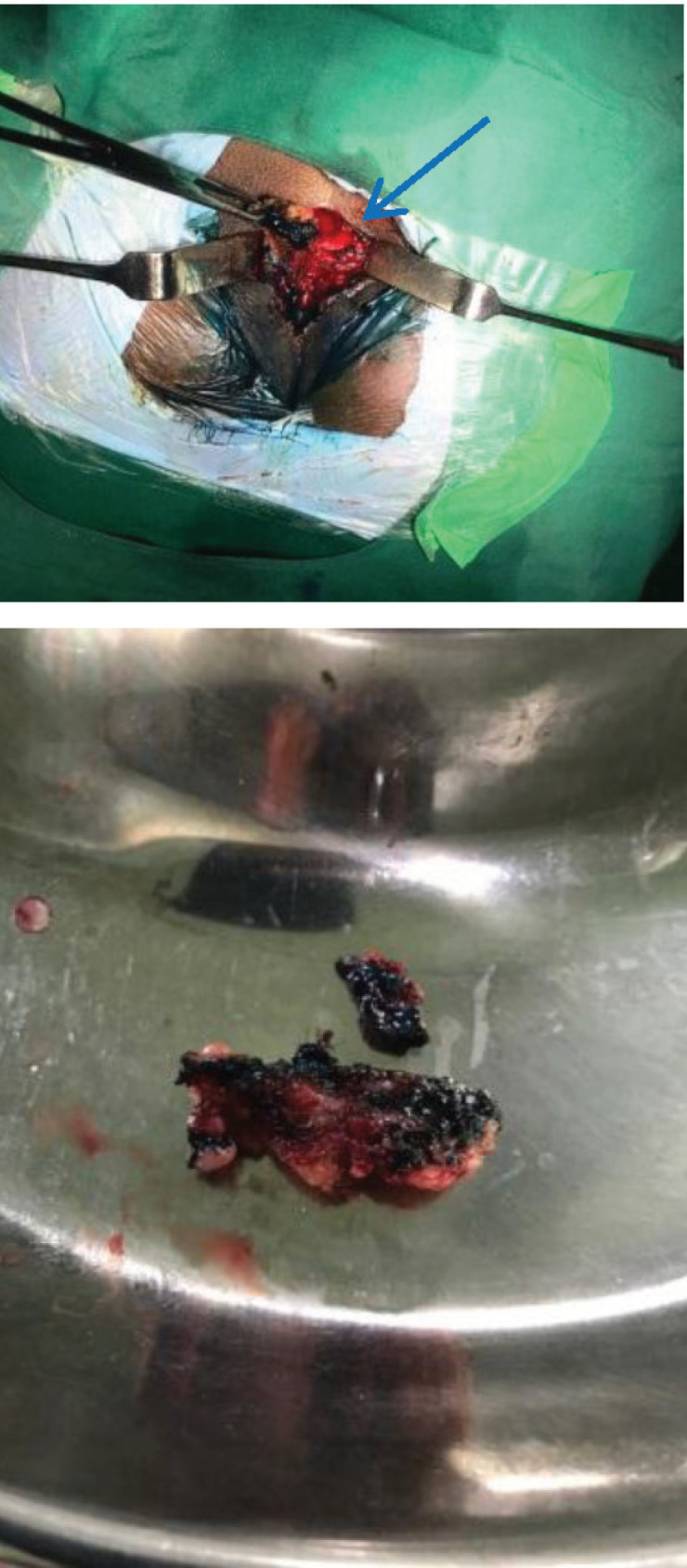
Appearance at surgery and excised material from sinus tract

The patient underwent successful excision of the sinus tract which contained dermoid material with hairs (Figure 4 and 5). Histopathological examination of the excised tissue showed fibrocollagenous and adipose tissue with congested blood vessels; there were areas of haemorrhage and infiltration by acute and chronic inflammatory cells with few multinucleate giant cells but no evidence of granuloma all suggestive of non-specific inflammatory changes. Culture study was sterile.

At 8 weeks follow-up the boy was reported to be doing and the scar site has nicely healed. He was discharged to the care of the local physician.

## DISCUSSION

The case study described the challenges that non-specialist clinicians may face with presentations of SD affecting the sacrococcygeal region only. Our case showed recurrent sinus tract infection which should have triggered a referral from the local physician to a specialist centre to prevent recurrent episodes of infections and the associated morbidities. 

Cutaneous congenital abnormalities are relatively common in the sacrococcygeal area; 2% to 4% of children have intergluteal dorsal dermal sinuses in the perianal region generally referred to as pits or dimples.^3^ Simple intergluteal dorsal dermal sinuses without other cutaneous findings, and where the base of the tract is clearly visible, usually do not require radiological or surgical evaluation and treatment.^3^ However, cases where other cutaneous markers of OSD or neurological symptoms are present, radiological investigations and further specialist evaluation may be indicated.^3^

In a retrospective study over a 30-years period from the USA of 28 operated cases with dermal sinus tract 9 patients had lumbosacral tracts explored. The age at presentation was <1 year of age in 16 cases, and >1 year in 12 patients.^4^ Cutaneous findings (n=15) were the main reason for referral, and infection in 3 cases.^4^

In a study from Iran of 35 children who were treated for spinal dermal sinus tract, the age range was between 3 days to 8.44 years and the lesions were located most frequently in the lumbar and lumbosacral regions.^5^ Abnormal skin findings (57.1%) and infection (31.4%) were the most common reasons; although 8/35 presented with meningitis.^5^ Neurologic abnormalities were detected in 37.1% cases, four of whom presented acutely with rapidly progressive paraplegia and meningitis.^5^ Tethered cord (63%) was the most common MRI finding in this cohort.^5^ All patients underwent complete resection of the tract and repair of associated abnormalities and none demonstrated any neurological deterioration postoperatively.^5^

A study from India with 21 children (male: female ratio of 13:8) treated for spinal dorsal dermal sinus, most cases (n=15) were aged between 2 and 15 years (mean age = 8.2 years).^6^ Lumbar region was the most frequently involved (11 cases) followed by thoracic (n=4), lumbosacral (n=3) and cervical (n=3).^6^ All children had neurological deficits detected including 3/21 with acute meningitis who had acute onset paraplegia and intraspinal abscess.^6^

Lack of awareness of the varied presentations of SD is one of the important reasons for delayed presentation and diagnosis^2^. In a multi-centre study involving 9 patients from the UK and India, the age at diagnosis varied from 2.5 months to 19 years.^2^ The mean duration from first assessment by a medical practitioner (based in the primary care set-up) to correct diagnosis and referral to a neurosurgeon was 5-years (range 2 months to 18 years).^2^

MRI remains the investigation of choice and it is vital that the neurosurgeons liaise with an experience radiologist to conduct and report the findings. In a recent study from India with 50 children with SD, 34% had type II Arnold-Chiari malformation, spina bifida occulta (22%) and diastematomyelia (18%) were commonly identified anomalies.^1^ MRI findings correlated well with surgical findings in the 20 operated cases where records were available. The author suggests that MR myelographic 3D-HASTE and STIR sequences should be a part of protocol to evaluate SD.^1^

## In conclusion, 

the boy in our case had all the features of OSD with a sinus tract, however delay in diagnosis was primarily due to lack of awareness in the physician treating the patient. The case study also reflects the unexpected diagnostic challenges that may be faced by clinicians working in resource-limited settings where advanced radiological imaging such as MRI scan may not be available in smaller centres. Practitioners should not ignore a midline visible sinus in the gluteal cleft. To facilitate an early diagnosis and referral to specialist services, primary care physicians need to be remain aware about congenital spinal dermal sinuses and their potential associations. 
